# Impact of Angiotensin-Converting Enzyme Inhibitors/Angiotensin Receptor Blockers on Renal Function in Chronic Kidney Disease Patients Undergoing Coronary Angiography

**DOI:** 10.7759/cureus.12808

**Published:** 2021-01-20

**Authors:** Arunee T Motes, Praveen Ratanasrimetha, Sariya Wongsaengsak, Yuttiwat Vorakunthada, Thammasak Mingbunjerdsuk, Camillo Pena, Kenneth Nugent

**Affiliations:** 1 Internal Medicine, Texas Tech University Health Sciences Center, Lubbock, USA; 2 Internal Medicine/Nephrology, University of Pittsburgh Medical Center, Pittsburgh, USA; 3 Internal Medicine/Pulmonary Medicine, Critical Care and Sleep Medicine, University of Arizona College of Medicine - Phoenix, Phoenix, USA; 4 Internal Medicine/Gastroenterology, University of Kentucky College of Medicine, Lexington, USA; 5 Internal Medicine/Nephrology, Texas Tech University Health Sciences Center, Lubbock, USA; 6 Internal Medicine/Pulmonary and Critical Care Medicine, Texas Tech University Health Sciences Center, Lubbock, USA

**Keywords:** angiotensin-converting enzyme inhibitors, angiotensin receptor blockers, contrast-induced acute kidney injury, coronary angiography

## Abstract

Background: Cardiac catheterizations and coronary angiography are minimally invasive methods for studying the heart and the coronary arteries, using iodinated radiocontrast agents which can cause acute kidney injury (AKI). Angiotensin-converting enzyme (ACE) inhibitors and angiotensin receptor blockers (ARB) are widely used due to their well-established benefit in coronary artery disease and renal protection in diabetes mellitus. Renin-angiotensin-aldosterone system inhibitors can induce AKI in some patients.

Method: This study analyzed the effect of radiocontrast media used for coronary angiography on renal function in patients with chronic kidney disease (CKD) stages 2-5 who also took ACE inhibitors/ARB medications. Information was collected from the electronic medical records of 116 cases to determine changes in serum creatinine following angiography.

Result: The average age of patients was 65.2 ± 12.3 years. There were 89 men (76.7%) and 27 women (23.3%). Six patients had documented ACE inhibitor discontinuation, and one patient had documented ARB discontinuation prior to their procedures. Based on the criteria of an increase in serum creatinine (SCr) by ≥0.3 mg/dl within 48 hours, 19 cases (16.4%) had AKI. Based on the criteria of increasing in SCr to ≥ 1.5 times baseline, AKI developed in 2 cases (1.7%) on day 1, 4 cases (3.5%) on day 2, and 7 cases (6.0%) on day 3 after coronary angiography.

Conclusion: This study suggests that the continuation of ACE inhibitors/ARB does not appear to have any important effect or association with changes in renal function, within one-month post angiography in patients with CKD stages 2-5.

## Introduction

More than a million cardiac catheterizations and coronary angiographies are performed every year in the United States. These are minimally invasive methods for studying the heart and the coronary arteries using iodinated radiocontrast agents that can cause acute kidney injury (AKI) [1]. Contrast-induced AKI is defined as any case of AKI occurring 48-72 hours after the intravascular injection of contrast media that cannot be attributed to other causes. It occurs more frequently in older patients, in patients with diabetes, and in patients with underlying renal insufficiency [2]. Contrast-induced nephropathy is a major cause of new-onset renal failure in hospitalized patients, and increased hospital length of stay and in-hospital mortality are directly related to the severity of contrast-induced nephropathy [3,4]. The reported rate of AKI after coronary catheterization ranges between 2% and 25% [5,6]. Patients with contrast-induced nephropathy who require dialysis have an in-hospital mortality rate close to 40% and two-year survival of less than 20% [7,8].

The reported risk of contrast-induced AKI is much higher with procedures involving the arterial administration of contrast, e.g., coronary angiography, than the venous administration of contrast. This difference in risk may be due to differences in patient populations since the patients who require arterial contrast are more likely to have comorbidities that increase the likelihood of developing AKI or due to differences in the nephrotoxicity associated with the intra-arterial administration of contrast in comparison to the intravenous administration of contrast [1].

Angiotensin-converting enzyme (ACE) inhibitors and angiotensin receptor blockers (ARB) are widely used for their well-established benefit in reducing morbidity and mortality in patients with coronary artery disease and for renal protection in patients with diabetes mellitus. ACE inhibition can induce AKI in patients in whom glomerular filtration is critically dependent on angiotensin II-mediated efferent vascular tone, such as in patients with renal arterial disease or in patients with heart failure and severe volume depletion [9]. Despite the fact that ACE inhibition can contribute to AKI in certain conditions, it remains controversial whether ACE inhibitor or ARB medications should be discontinued prior to coronary angiography to minimize post-procedure AKI. According to the Kidney Disease-Improving Global Outcomes guidelines for AKI, there is insufficient evidence to recommend discontinuation of these drugs in patients undergoing injection of contrast drugs [10].

In this study, we wanted to determine if radiocontrast exposure during coronary angiography causes deterioration in renal function in patients with CKD stages 2-5 who are not on dialysis and who take ACE inhibitor/ARB medications. These patients are often older and have comorbidity relevant to renal disease. We investigated the incidence of AKI after coronary angiography in CKD patients taking ACEI/ARB medication during the 30 days prior to the procedure.

## Materials and methods

We included patients with CKD stages 2-5 (estimated glomerular filtration rate [eGFR] < 90 ml/min) who took ACE inhibitor/ARB medications ≥1 month before coronary angiography and were evaluated in our hospital between January 1, 2015, and December 31, 2016. We excluded CKD patients who were less than 18 years old, pregnant, on dialysis, or kidney transplant recipients. The eGFR in our hospital laboratory is based on the Modification of Diet in Renal Disease (MDRD) Study equation. This study was approved by the Institutional Review Board at Texas Tech University Health Sciences Center (L18-079) in Lubbock, Texas.

A total of 116 cases were included in the study. From electronic medical records, we collected information on gender, age, cardiac disorders, comorbidities, diabetes mellitus, hypertension, the presence of proteinuria, amount of proteinuria in spot urine samples, CKD stages 2-5, ACE inhibitor medication, ACE inhibitor medication discontinuation, ARB medication, ARB medication discontinuation, non-steroidal anti-inflammatory drugs use, date of coronary angiography, serum creatinine (SCr)/eGFR before coronary angiography, SCr/eGFR on day 1 after coronary angiography, SCr/eGFR on day 2 after coronary angiography, SCr/eGFR on day 3 after coronary angiography, SCr/eGFR one month after coronary angiography, length of hospital stay, rehospitalization, repeat coronary angiography, and cardiovascular complications. Patients undergoing cardiac catheterization and angiography in our cardiac catheterization lab receive individualized periprocedural fluid management; there is no standard fluid protocol. A low osmolar contrast agent, such as Visipaque (VISIPAQUE™ (iodixanol) injection | GE Healthcare, Chicago, IL), was used in the catheterization procedures. This lab did not consistently record contrast volumes. There was also limited information in the medical records about the discontinuation of the ACE inhibitor/ARB medications. The catheterization lab does not have a consistent policy regarding the use of these medications prior to catheterization. Consequently, it was difficult to determine which patients had the medications discontinued and who did not and the timeframe for any discontinuation; therefore, the collected data were interpreted as if the patients continued to take ACE inhibitor/ARB medications prior to the procedure. This lab did not consistently record contrast volumes.

## Results

The average age of patients was 65.2 ± 12.3 years (Table 1). There were 89 men (76.7%) and 27 women (23.3%). Among the 116 cases, six cases had documented ACE inhibitor discontinuation, and one case had documented ARB discontinuation prior to the procedure. Twenty-nine cases had CKD stages 4 and 5 prior to coronary angiography; 44 cases had proteinuria with spot urine protein levels varying from 30 mg/dl to more than 600 mg/dL. There were 76 patients (65.5%) who had diabetes mellitus and 97 patients (83.6%) who had hypertension. Thirteen cases underwent repeat coronary angiography, and 12 cases developed cardiovascular complications, including cardiogenic shock, arrhythmia, cardiac arrest, and heart failure. Fifteen patients required rehospitalization for any cause after the coronary angiography.

**Table 1:** Demographic data ACEI: angiotensin-converting enzyme inhibitor, ARB: angiotensin receptor blocker, CKD: chronic kidney disease, NSAID: nonsteroidal anti-inflammatory drug.

Parameter	Number	Percentage (%)
Gender		
Male	89	76.72
Female	27	23.28
Age (years)		
18-20	0	0.00
21-30	0	0.00
31-40	3	2.59
41-50	14	12.07
51-60	24	20.69
61-70	32	27.59
71-80	34	29.31
81-90	7	6.03
>90	2	1.72
Cardiac disorders		
Coronary artery disease	71	61.21
Heart failure	26	22.41
Atrial fibrillation	8	6.90
Comorbidity		
Diabetes mellitus	76	65.52
Hypertension	97	83.62
CKD stage 4-5	29	25.00
Proteinuria	44	37.93
NSAID use	4	3.45
ACEI		
Lisinopril	91	78.45
Enalapril	5	4.31
Captopril	6	5.17
Ramipril	3	2.59
ARB		
Losartan	3	2.59
Valsartan	4	3.45
Candesartan	3	2.59
Olmesartan	1	0.86

Based on the criterion of increasing SCr by ≥0.3 mg/dl within 48 hours, 19 cases (16.4%) had AKI. Based on the criterion of increasing SCr to ≥ 1.5 times baseline, which was available within the prior seven days, AKI was diagnosed in two cases (1.7%) on day 1, in four cases (3.5%) on day 2, and in seven cases (6.0%) on day 3 after coronary angiography. Urine volumes were not measured after coronary angiography.

Table 2 reports the average SCr and eGFR. The average SCrs in mg/dL were 2.30, 1.97, 2.00, 1.93, and 2.21 at baseline, day 1, day 2, day 3, and one month after coronary angiography, respectively. Average eGFRs in ml/minute were 44.17, 43.70, 41.83, 43.58, and 42.88 at baseline, day 1, day 2, day 3, and 1 month after coronary angiography, respectively. The mean eGFR in patients classified by CKD stage are reported in Table 3. Eighty-three cases out of 116 cases had documented SCr at one month. Post-procedure, thirty-five cases (42.2% of 83) had increased SCr at one month compared to their baseline, 35 cases (42.2%) had decreased SCr compared to their baseline, and 13 cases (15.6%) had unchanged SCr compared to their baseline.

**Table 2:** Average SCr and eGFR before and after coronary angiography SCr: serum creatinine, eGFR: estimated glomerular filtration rate.

	Baseline	Day 1	Day 2	Day 3	1 month
Average SCr (mg/dl)	2.03 ± 1.54	1.97 ± 1.43	2.0 ± 1.20	1.93 ± 1.17	2.21 ± 1.69
Average eGFR (ml/min)	44.17 ± 18.92	43.7 ± 18.03	41.83 ± 18.80	43.58 ± 19.16	42.88 ± 21.58

**Table 3:** Estimated GFR before and after angiography in each stage of CKD CKD: chronic kidney disease, eGFR: estimated glomerular filtration rate, PCI: percutaneous coronary intervention.

CKD stage	Number	Average eGFR ± SD				
		Before PCI	Day 1	Day 2	Day 3	1 month
2	22	68.73±7.57	62.48±11.78	60.7±15.17	62.4±13.85	62.81±16.55
3a	40	52.22±4.82	51.99±10.47	47.49±16.66	45.97±17.68	54.04±13.79
3b	25	39.5±4.75	42.62±9.54	40.39±10.80	42.15±12.85	38.99±9.91
4	15	24.88±3.30	25.23±6.63	25.28±8.49	27.53±10.84	28.24±21.35
5	14	11.12±3.24	12.56±4.78	16.3±11.14	16.35±8.95	12.82±8.25

In the ACE inhibitor/ARB discontinuation group (n=7), the mean eGFR ± SD in ml/minute were 36.00 ± 12.06, 37.92 ± 10.94, 35.11 ± 8.03, 35.02 ± 7.56, and 35.21 ± 5.70 before the procedure, at day 1, day 2, day 3, and one month after coronary angiography, respectively.

## Discussion

This study revealed that the continuation of the ACE inhibitor/ARB medications is not associated with significant renal injury in older patients with CKD stages 2-5 baseline renal disease undergoing coronary angiography. These results suggest that patients can continue ACE inhibitor/ARB medications during and after cardiac catheterization procedures provided that no intercurrent illness dictates an alternative plan.

Angiotensin II is a potent vasoconstrictor of the systemic and renal vascular beds. Consequently, ACE inhibitors cause systemic and renal vasodilation, resulting in a fall in blood pressure and an increase in renal blood flow. As renal vasodilation mainly occurs in the efferent arteriole, glomerular filtration pressure is reduced by ACE inhibition. Simultaneously, angiotensin II induces mesangial cell contraction, resulting in an increase in the surface area available for filtration. As a consequence, the decreased filtration pressure does not cause a reduction in glomerular filtration rate, as it is counterbalanced by the simultaneous increases in renal blood flow and glomerular filtration area. However, in conditions in which glomerular filtration is critically dependent on angiotensin II-mediated efferent vascular tones, such as with a post-stenotic kidney or with heart failure and severe volume depletion, ACE inhibition can induce AKI, which is usually reversible after withdrawal of the drug [9].

A recent systematic review and meta-analysis study that included three randomized controlled trials (RCTs) and three prospective cohort studies (1663 total participants) revealed that there is low-quality evidence that demonstrates that withdrawal of ACE inhibitor/ARB medications prior to coronary angiography and cardiac surgery reduces the incidence of AKI and there was no evidence that drug discontinuation reduces the incidence of AKI during intercurrent illness in primary or secondary care [11].

Wolak et al. randomized patients into three groups, i.e., group A (ACE inhibitor/ARB stopped 24 hours prior to the procedure and restarted immediately after the procedure), group B (ACE inhibitor/ARB stopped 24 hours prior to the procedure and restarted 24 hours after the procedure), and group C (ACE inhibitor/ARB continued throughout the study period) (total N=94) and concluded that ACE inhibitors and ARB medications can be safely used before and after coronary angiography in patients with an eGFR ≥ 60 ml/min. However, post-hoc analysis in this study suggested that it might be advisable to discontinue ACEI/ARB at least 24 hours before coronary angiography in patients with eGFR < 60 ml/min [12]. An RCT with 220 patients reported that withholding ACE inhibitor and ARB medications 24 hours before coronary angiography does not alter the incidence of contrast-induced nephropathy in stable patients with CKD stages 3-4 (eGFR 15-60 ml/min) [13]. Bainey et al. demonstrated that in patients (N=208) with moderate renal insufficiency undergoing cardiac catheterization withholding ACE inhibitor/ARB medications resulted in a non-significant reduction in contrast-induced AKI and a significant reduction in the post-procedural rise of creatinine. This low-cost intervention could be considered when referring a patient for cardiac catheterization [14]. These studies are summarized in Table 4.

**Table 4:** Details of studies included in the review SCr: serum creatine, AKI: acute kidney injury, CKD: chronic kidney disease, ACEI: angiotensin-converting enzyme inhibitor, ARB: angiotensin receptor blocker, eGFR: estimated glomerular filtration rate, A-fib: atrial fibrillation, CIN: contrast-induced nephropathy, RCT: randomized controlled trial.

	Our study	Wolak et al. [12]	Rosenstock et al. [13]	Bainey et al. [14]
Study design	Retrospective	RCT	RCT	RCT
Time period	January 2015 - June 2017	April 2010 - September 2010	No details	July 2006 - March 2012
Sample size	116	94	220	208
Mean age	65 ± 12	65 ± 12	72 ± 10	73 ± 9
Female (%)	23	33	52	26
Risk group	CKD stages 2-5	None	CKD stages 3-4	CKD stage 3
Baseline SCr	2.03 ± 1.5	1.01 ± 0.4	1.5 ± 0.4	1.6 ± 0.4
Comorbidities	Hypertension (83.6%), diabetes (62.5%), heart failure (22.4%), A-fib (6.9%)	Unstable angina (62%), diabetes (50%)	Hypertension (97%), diabetes (54%)	Diabetes (54%), hypertension (47%), heart failure (14%)
AKI definition	Increase in SCr by ≥0.3 mg/dl within 48 hours or increase in SCr to ≥1.5 times baseline	Increase in SCr by ≥25% from baseline	Increase in SCr by >25 % or ≥0.5 mg from baseline	Increase in SCr by ≥25% or ≥0.5 mg from baseline
Study drug	ACEI/ARB	ACEI/ARB	ACEI/ARB	ACEI/ARB
Time of hold	Insufficient data	24 hours prior to the procedure	Day of procedure	24 hours prior to the procedure
Procedure	Coronary angiogram	Coronary angiogram	Coronary angiogram	Coronary angiogram
Study outcome	There is no definite evidence to indicate that angiography causes AKI in CKD stages 2-5 patients who take ACEI/ARB.	ACEI and ARB can safely be used before and after angiography in patients with eGFR ≥ 60 ml/minutes	Withholding ACEIs and ARBs 24 h before angiography does not alter the incidence of CIN in stable patients with CKD stages 3–4.	Withholding ACEI/ARB resulted in a non-significant reduction in contrast-induced AKI and a significant reduction in the post-procedural rise of creatinine.

Mehran et al. have reviewed contrast-induced nephropathy and discussed the possibility that avoiding needed contrast studies might do more harm than undertaking the studies in some patients [15]. Therefore, patient evaluation requires careful consideration of the risks and benefits associated with the procedure. In addition, the best method for reducing renal injury involves the use of intravenous saline before and after the procedure, but the best protocol for this is uncertain.

Our study has several limitations. First, in a retrospective study, it is difficult to determine causal relationships in complicated patients. Second, there was limited information in the medical records about ACE inhibitor/ARB medication discontinuation. The catheterization lab does not have a consistent policy regarding the use of these medications prior to catheterization. Consequently, it was difficult to determine which patients had the medications discontinued and which patients did not and the timeframe for any discontinuation. Therefore, the collected data were interpreted as if the patients continued to take ACE inhibitor/ARB medications prior to the procedure. We did not find any significant difference between baseline average SCr and average SCr at 48 hours after the procedure, and the average SCr at 72 hours was, in fact, lower than baseline average SCr. Third, the records did not provide consistent information on the volume of contrast use during procedures. Future studies should include the timeframe for the discontinuation of the ACE inhibitor/ARB medication, and information about the blood pressures prior to and after cardiac catheterization.

## Conclusions

This study suggests that the continuation of ACE inhibitor/ARB medications does not appear to have any important effect or association with changes in renal function within one month post angiography in patients with CKD stages 2-5. This information should help hospitalists and cardiologists schedule coronary catheterization studies with the most efficient use of hospital time. Of course, all patients with chronic renal disease need careful clinical assessment before any procedure involving catheterization and contrast injection.
